# In-Depth Sequence Analysis of Bread Wheat *VRN1* Genes

**DOI:** 10.3390/ijms222212284

**Published:** 2021-11-13

**Authors:** Beáta Strejčková, Zbyněk Milec, Kateřina Holušová, Petr Cápal, Tereza Vojtková, Radim Čegan, Jan Šafář

**Affiliations:** 1 Centre of the Region Haná for Biotechnological and Agricultural Research, Institute of Experimental Botany of the Czech Academy of Sciences, 77900 Olomouc, Czech Republic; strejckova@ueb.cas.cz (B.S.); milec@ueb.cas.cz (Z.M.); holusovak@ueb.cas.cz (K.H.); capal@ueb.cas.cz (P.C.); vojtkova@ueb.cas.cz (T.V.); cegan@ibp.cz (R.Č.); 2 Department of Cell Biology and Genetics, Faculty of Science, Palacký University, 78371 Olomouc, Czech Republic; 3 Department of Plant Developmental Genetics, Institute of Biophysics of the Czech Academy of Sciences, 61200 Brno, Czech Republic

**Keywords:** *VRN1*, allelic variation, wheat, CNV, next generation sequencing, alternative splice variants

## Abstract

The *VERNALIZATION1* (*VRN1*) gene encodes a MADS-box transcription factor and plays an important role in the cold-induced transition from the vegetative to reproductive stage. Allelic variability of *VRN1* homoeologs has been associated with large differences in flowering time. The aim of this study was to investigate the genetic variability of *VRN1* homoeologs (*VRN*-*A1*, *VRN*-*B1* and *VRN*-*D1*). We performed an in-depth sequence analysis of *VRN1* homoeologs in a panel of 105 winter and spring varieties of hexaploid wheat. We describe the novel allele *Vrn*-*B1f* with an 836 bp insertion within intron 1 and show its specific expression pattern associated with reduced heading time. We further provide the complete sequence of the *Vrn*-*A1b* allele, revealing a 177 bp insertion in intron 1, which is transcribed into an alternative splice variant. Copy number variation (CNV) analysis of *VRN1* homoeologs showed that *VRN*-*B1* and *VRN*-*D1* are present in only one copy. The copy number of recessive *vrn*-*A1* ranged from one to four, while that of dominant *Vrn*-*A1* was one or two. Different numbers of *Vrn*-*A1a* copies in the spring cultivars Branisovicka IX/49 and Bastion did not significantly affect heading time. We also report on the deletion of secondary structures (G-quadruplex) in promoter sequences of cultivars with more *vrn*-*A1* copies.

## 1. Introduction

Bread wheat (*Triticum aestivum* L., 2*n* = 6*x* = 42) is one of the most important crops worldwide. It originated in the Fertile Crescent via hybridization of tetraploid and diploid ancestors and was domesticated in this region. As human civilization expanded, wheat cultivation spread to both hemispheres, which was facilitated by its ability to adjust its flowering time in response to different growing conditions [1]. The overall flowering pathway includes the photoperiod response associated with *PHOTOPERIOD1* (*PPD1*) genes [2,3,4], and the vernalization pathway, which is associated with the cold-induced transition from the vegetative to reproductive stage. The *VERNALIZATION1* (*VRN1*) gene encoding a MADS-box transcription factor (TF) expressed in leaves and the shoot apical meristem plays a significant role in the vernalization response [5,6]. Other vernalization genes, such as *VRN2* and *VRN3*, are also important members of the flowering pathway. *VRN2* encodes a long-day dominant repressor of flowering while *VRN3* encodes a mobile protein operating flowering activator [7,8,9].

In winter wheat carrying an intact *VRN1* gene, exposure to low temperature for a certain period of time (vernalization) accelerates flowering [10]. Indels within the promoter region of *VRN1*, or a deletion in its first intron, are typical for dominant alleles and lead to a high basal level of *VRN1* expression, resulting in a spring growth habit [11,12]. The first intron contains the specific sequence motif RIP3, a putative binding site for the flowering repressor *TaGRP2* [13]. Based on the RIP3 sequence motif, RIP3 1_SNP and RIP3 3_SNP haplotypes have been described [14]. Dominant *Vrn1* alleles present in spring wheats either show large deletions in the first intron removing this binding site or have a mutation in the promoter region.

To date, several *VRN1* alleles have been described. The *Vrn-A1a* allele, which prevails in hexaploid spring wheat accessions, includes a duplicated region with a mutator DNA transposon DTM_Spring_TREP1674-1 (“spring” foldback element, SFE) inserted into the promoter [15]. Other known alleles with altered promoters include *Vrn*-*A1B* [15] and *Vrn*-*D1c* [16], which contain deletions and insertions, respectively. The most frequent mutation within the first *VRN1* intron is a deletion of variable length present in the *Vrn*-*A1c*, *Vrn*-*A1iAUS*, *Vrn*-*B1a*, *Vrn*-*B1b*, *Vrn*-*B1c*, *Vrn*-*D1a* and *Vrn*-*D1b* alleles [12,17,18,19,20,21]. Transposable element (TE) insertion within the first intron was described in *Triticum spelta* (L.) and designated *Vrn-D1s* [22].

Copy number variation (CNV) is an important type of structural genome variation that contributes to phenotype plasticity in plants [23,24,25,26]. In bread wheat, CNV in recessive as well as dominant *VRN1* alleles has been reported [4,27,28,29]. The wheat *Vrn*-*D4* gene, which confers a spring phenotype, may be considered a special case of CNV. It is characterized by the insertion of an ≈290-kb duplicated region from chromosome 5AL (including *vrn*-*A1*) into chromosome 5DS. *Vrn-D4* can be found in the majority of spring wheat accessions in South Asia [30].

In wheat, vernalization requirements range from three to eight weeks [31,32]. This difference has been attributed to two *VRN1* single nucleotide polymorphisms (SNPs) resulting in either an amino acid change or the removal of a flowering repressor binding site [14,31]. Stronger vernalization requirements and late flowering have also been linked with increased copy numbers of the *vrn*-*A1* gene [4].

Other potential mechanisms for the modulation of the vernalization response include DNA methylation and post-translational modification. Vernalization-induced hypermethylation at specific non-GC sites in TE fragments located within the first intron has been reported, although its contribution to *VRN1* regulation needs to be further investigated [33]. Alonso-Peral et al. [11] suggested a model for transcriptional regulation of *VRN1*, where the locus is maintained in an inactive state prior to winter by an H3K27me3 mark deposited by the Polycomb Repressive Complex 2 (PRC2), whose core components were recently identified in bread wheat [34]. While no significant changes at the H3K27me3 level were detected in the *vrn*-*A1* promoter after vernalization treatment [35], a shift from H3K27me3 to H3K4me3 during vernalization was reported within the “critical” region of the first intron [13].

In this study, we sequenced complete *VRN*-*A1*, *VRN*-*B1* and *VRN*-*D1* genes, including their promoter regions, in 65 winter wheat cultivars and 40 spring wheat cultivars. We report the sequence of a novel dominant allele as well as its transcription profile. Moreover, we provide a protocol for copy number determination of *VRN*-*B1* and *VRN*-*D1* homoeologs using droplet digital PCR (ddPCR). The vernalization response is quantitative in nature; therefore, the combination of individual *VRN1* homoeologous alleles can significantly affect the heading date. A detailed characterization of *VRN1* alleles may be beneficial for breeding climate-resilient wheat cultivars.

## 2. Results

### 2.1. CNV of VRN1 Homoeologs

CNV of *VRN*-*A1*, *VRN*-*B1* and *VRN*-*D1* was estimated in all 65 winter and 40 spring cultivars (Supplementary Table S1). The presence of *VRN*-*A1* copies was determined using the *VRN*-*A1* TaqMan^®^ assay following Díaz et al. (2012). To estimate the CNV of *VRN*-*B1* and *VRN*-*D1*, we developed a ddPCR assay, as described in the Materials and Methods section (Table 1). Screening using published primer pairs [12,15,19] revealed that 14 of the 40 spring cultivars carried recessive *vrn*-*A1*, 23 cultivars carried dominant *Vrn*-*A1a*, 2 cultivars carried dominant *Vrn*-*A1b*, and the remaining cultivar carried a combination of the dominant alleles *Vrn*-*A1b* and *Vrn*-*D4*. CNV was detected for both recessive and dominant alleles (Table 2).

While the copy number for the *vrn*-*A1* allele varied from one to four copies, dominant *Vrn*-*A1a* was present in one or two copies, and dominant *Vrn*-*A1b* was present in two copies exclusively. One copy of recessive *vrn*-*A1* was detected in only 4 of the 65 winter wheats (6%). Two copies were present in eighteen winter cultivars (28%), and three copies were the most frequent state, detected in forty-one winter wheat cultivars (63%). Four copies were observed in only two winter cultivars (3%). Twenty-three spring cultivars carried either one or two copies of the dominant *Vrn*-*A1a* allele. Dominant *Vrn*-*A1b* present in two copies was detected in three varieties. In contrast to the *VRN*-*A1* homoeolog, which showed a variable copy number, only one copy each of *VRN*-*B1* and *VRN-D1* was detected in each of the cultivars.

### 2.2. VRN1 Sequence Variability and Gene Expression

Multiple alignments of *VRN1* sequences from the panel of 105 cultivars with the reference sequence revealed high sequence similarity overall (SM1). The most variable region of the gene appeared to be the first intron, which included the majority of the detected SNPs and several new insertions and deletions.

#### 2.2.1. Sequence Analysis of *VRN*-*A1* Genes and Promoters

**Figure 1:**
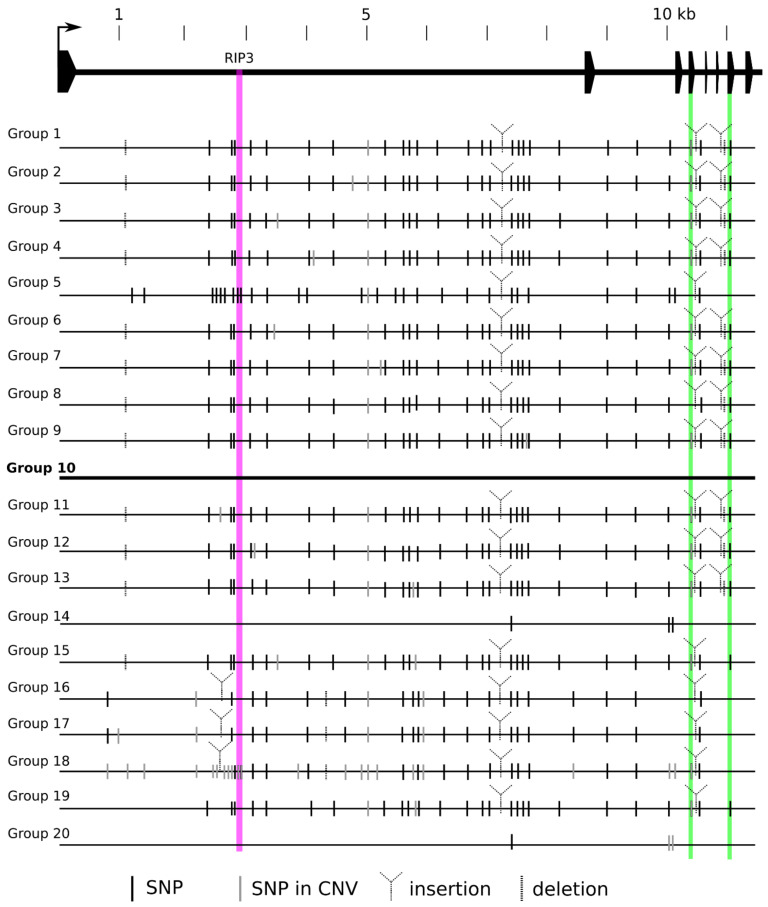
Nucleotide polymorphism of 20 *vrn*-*A1* haplotypes. SNPs and indels revealed by sequence analysis of the complete *vrn*-*A1* gene from 105 bread wheat varieties. SNP means polymorphism in comparison to Triple Dirk C; SNP in CNV means polymorphism among individual copies within the same variety. Varieties sharing the same SNP pattern are grouped together. The structure of the *vrn*-*A1* gene is shown at the top of the scheme. All variations are based on comparison with Group 10 (containing Triple Dirk C), which was set as the reference. Purple vertical bar represents the RIP3 binding site; green vertical bars represent exon 4 and exon 7. Detailed information is provided in Supplementary Table S2.

Among the 105 cultivars, 79 carried recessive *vrn*-*A1*, 23 carried dominant *Vrn*-*A1a*, and 3 carried the dominant *Vrn*-*A1b* allele. The spring cultivar VL-30 possesses the *Vrn*-*D4* allele together with two copies of the dominant *Vrn*-*A1b* allele. Based on *VRN*-*A1* Illumina sequence of similarity and the presence of SNPs, the cultivars were divided into 20 groups (Supplementary Table S2). The sequence variation of *VRN*-*A1* haplotype groups is depicted in Figure 1. The largest groups, Groups 1 and 14, comprised 23 wheat cultivars each. Cultivars with one copy of recessive *vrn*-*A1* were divided into two groups: 5 and 10. Group 5 contained all cultivars with the RIP3 3_SNP haplotype, differing at several SNPs from Group 10, which included cultivars with the RIP3 1_SNP haplotype, including the reference line Triple Dirk C (TDC). The diagnostic A367C SNP in exon 4 of VL-30 (Group 18) indicated that one of the three copies is actually the dominant *Vrn*-*D4* allele [30].

**Figure 2:**
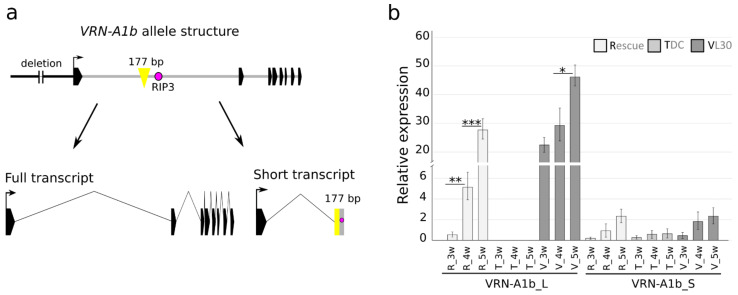
The 177 bp insertion found in *Vrn*-*A1B* intron 1 is transcribed as part of the alternative splice variant *VRNA1*-short. (**a**) Schematic representation of the *Vrn*-*A1B* allele with a 177 bp insertion within the first intron and *Vrn*-*A1b* splice variants. The full transcript (*VRN*-*A1b*_L) corresponds to the eight exons, and the alternative splice variant *VRNA1*-short (*VRN-A1b*_S) contains the first exon fused to part of the first intron, including the 177 bp insertion and RIP3. (**b**) Mean transcript levels (three biological replications) of two *vrn*-*A1* alternative splice variants in the spring cultivars Rescue with *Vrn*-*A1b* and VL-30 with *Vrn*-*A1b* and *Vrn*-*D4* and the winter cultivar TDC with intact *vrn*-*A1;* non-vernalized plants were sampled from 1 week old, 3 week old and 5 week old plants. * *p* value < 0.05, ** *p* value < 0.01, *** *p* value < 0.001, significance determined by paired Student’s *t*-test.

A 177 bp insertion in the first intron of the *Vrn*-*A1b* allele was found in the cultivars Pyrothrix 28, Rescue and VL-30 (Figure 2a). The insertion can be identified by PCR with the specific diagnostic primers VRNA1_177inF/R (Supplementary Table S3). The amplicon size is 854 bp, while the intact *vrn*-*A1* allele produces a 677 bp amplicon. In the cultivar VL-30, we detected PCR products corresponding to two identical copies of *Vrn*-*A1b* (854 bp) and one copy of *Vrn*-*D4* (677 bp) (Supplementary Figure S1). The insertion was also found in the wild emmer wheat cultivars carrying the *Vrn*-*A1b* allele (data not shown), indicating its conserved origin. Xiao et al. (2014) described an alternative splice variant, “*VRNA1*-short”, in which the first exon merged with part of the first intron, including the RIP3 region. We show that the 177 bp long insertion found in the *Vrn*-*A1b* genomic sequence is also present in the *Vrn*-*A1b* alternative short transcript (Figure 2a, Supplementary Figure S2) and can be amplified from cDNA with the diagnostic primers VRNA1S_177inF/R (Supplementary Table S3). Sequence variants of the *Vrn*-*A1b* short transcript are presented in Supplementary Figure S3.

The expression of both full and short *vrn*-*A1* transcripts in winter wheat cultivars with recessive *vrn*-*A1* was analyzed by Kippes et al. [14]. We used the same transcript-specific primers for expression analysis of the full and short *Vrn*-*A1b* transcripts (*Vrn*-*A1b*_L and *Vrn*-*A1b*_S, respectively) in the cultivars Rescue and VL-30. TDC (*vrn*-*A1*) was used to compare the abundances of *VRNA1*-short transcripts with and without 177 bp insertions. As expected, the non-vernalized winter line TDC had a significantly lower abundance of the full transcript than Rescue (*Vrn*-*A1b*) and VL-30 (*Vrn*-*A1b* and *Vrn*-*D4*). The short transcript was more abundant in spring cultivars. When analyzing the presence of the full-length and short transcripts in spring cultivars, the full transcript was more abundant, whereas in non-vernalized TDC, the short transcript was expressed at a higher rate than the full transcript as described by Kippes et al. [14] (Figure 2b).

**Figure 3:**
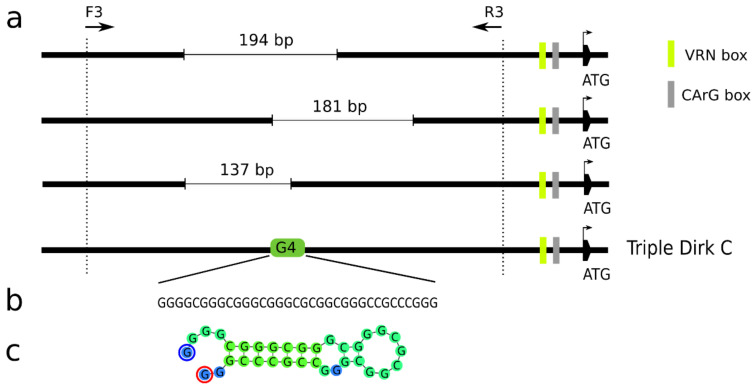
The secondary structure of the *vrn*-*A1* promoter may prevent successful amplification and sequencing. (**a**) Schematic representation of *vrn*-*A1* promoter variants with 137 bp, 181 bp and 194 bp deletions found in winter cultivars and the position of the G-quadruplex (G4). (**b**) Sequence motif of G4 found in Triple Dirk C (TDC, MH347747). (**c**) DNA fold prediction of G4 found in TDC.

By sequencing the *vrn*-*A1* promoter, several types of sequences amplified with the primers VRN1_prom_F3 and VRN1_prom_R3 [14] were obtained. The majority of the sequences were possible off-targets of the primer pair used. Only a small fraction of sequences corresponded to the known sequence of promoter *vrn*-*A1* of TDC (GenBank MH347747), but all of them contained partially overlapping deletions of different lengths (Figure 3a). In the cultivar Ludwig (three copies of *vrn*-*A1*), two variants with deletions of 137 bp and 181 bp were found. The same 181 bp long deletion was also detected in the cultivars Brokat, Batis, Banderola (three copies of *vrn*-*A1*) and Brilliant (two copies of *vrn*-*A1*). In the cultivar Kosutka (two copies of *vrn*-*A1*), a deletion of 194 bp was revealed (Figure 3a). A 34 bp long G-quadruplex located 784 bp upstream of the start codon (Figure 3b, Supplementary Table S4) of the intact *vrn*-*A1* allele of TDC was deleted in the abovementioned cultivars (Figure 3a), indicating that the intact *vrn*-*A1* sequence was not amplified and sequenced due to its stable secondary structure (Figure 3c) [37]. Otherwise, no additional sequence polymorphisms were found, in addition to known mutations distinguishing the recessive *vrn*-*A1* and dominant *Vrn-A1a* or *Vrn*-*A1b* alleles (SM1).

#### 2.2.2. Sequence Analysis of *VRN*-*B1* Genes and Promoters

**Figure 4:**
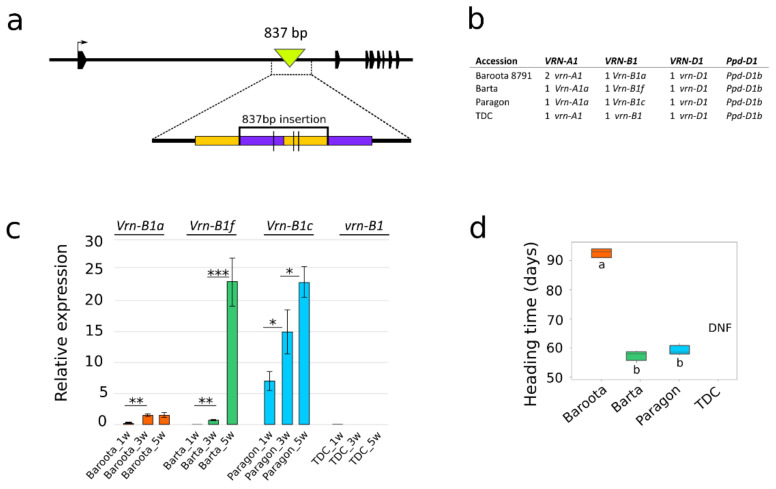
The *Vrn-B1f* allele with an 837 bp insertion affects heading time. (**a**) Schematic representation of the *Vrn*-*B1f* allele with an 837 bp insertion within the first intron. Polygons represent exons, inverted triangles represent insertions, duplicated regions share the same color, and vertical lines indicate SNPs. (**b**) Composition of the *VRN1* and *Ppd*-*D1* alleles. The number in front of *VRN1* alleles represents the number of copies. (**c**) Mean expression (three biological replicates) of different *VRN*-*B1* alleles in non-vernalized plants; samples were collected from 1-week-old, 3-week-old and 5-week-old plants; * *p* value < 0.05, ** *p* value < 0.01, *** *p* value < 0.001, significance determined by paired Student’s *t*-test. (**d**) Mean heading time of varieties with different *VRN*-*B1* alleles. Means that do not share the same letter are significantly different according to Tukey’s test (*p* < 0.05). TDC—Triple Dirk C, DNF—did not flower within 110 days.

Eighty of the 105 cultivars carry recessive *vrn-B1* alleles. Sequencing revealed 15 *vrn*-*B1* variants differing in SNPs (Groups 1B–15B in Supplementary Table S5). The dominant alleles *Vrn*-*B1a* and *Vrn*-*B1c* were present in 15 and 7 cultivars, respectively. The largest group was Group 1B, consisting of 53 winter and spring cultivars. The most variable sequence, with 45 detected intronic polymorphisms, including small indels and 36 bp long deletions within the first intron, was observed for Atlas 66 in Group 14B. A new allele (hereafter referred to as *Vrn*-*B1f*), defining Group 20B, was detected in three spring cultivars: Anza, Barta and Marquis. PCR amplification with the vrnB1_4F and vrnB1_4R primers [28] produced an ≈7-kb amplicon in Anza, Barta and Marquis (01C0201025) (Supplementary Figure S4), in contrast to the 6 kb amplicon in all other cultivars, including the reference TDC. Oxford Nanopore resequencing showed that compared with TDC, all three spring cultivars possessed an 837 bp insertion consisting of two duplicated regions (Figure 4a). We designed new primers to detect this insertion (Supplementary Table S3). This allele has been designated *Vrn*-*B1f* (GenBank accessions MZ593843, MZ593844 and MZ593845). To assess the influence of the new *Vrn*-*B1f* allele on heading time, TDC and three spring cultivars (Barta, Baroota 8791 and Paragon) carrying three different *VRN*-*B1* alleles, *Vrn*-*B1f*, *Vrn*-*B1a* and *Vrn*-*B1c*, respectively, were chosen for the heading time and RT–qPCR experiment (Figure 4b) with the designed q.VRNB1_F and q.VRNB1_R primers (Supplementary Table S6 and Figure S5). The expression analysis shows that the *Vrn*-*B1c* level significantly increases from week one to week five, whereas the level of *Vrn*-*B1a* increases only slightly and does not equal that of *Vrn*-*B1c*. In contrast, the level of *Vrn*-*B1f* rises very slowly in the first three weeks, with a sudden peak in the fifth week, nearly equaling that of *Vrn*-*B1c* (Figure 4c). The *Vrn*-*B1f* expression level was associated with altered heading time, with Barta heading approximately 30 days earlier (likewise for Paragon with *Vrn*-*B1c*) than Baroota 8791 and TDC failing to flower within 110 days (Figure 4d).

Then, we sequenced the 4.5 kb region upstream of the *VRN*-*B1* start codon. The nucleotide alignment of all 105 cultivars (SM1) showed that the VRN box and CArG box remained intact. All sequenced cultivars share a 30 bp long G4 motif located 735 bp upstream of the first exon. In 102 cultivars, a 23 bp long G4 situated 274 bp upstream of the start codon was also detected, but this feature was disrupted in the cultivars Atlas 66, Rumunka and 771-VII/12 (Supplementary Table S4, SM1). Overall, the *VRN*-*B1* promoter sequence is highly conserved (GenBank accession OK556477). Within the upstream sequence, we found 54 SNPs and 13 indels (1–7 bp). Most polymorphic promoters are found in the winter cultivars Atlas 66 and Rumunka and the spring cultivar 771-VII/12, which also show high *VRN*-*B1* gene polymorphism (Supplementary Table S5).

#### 2.2.3. Sequence Analysis of *VRN*-*D1* Genes and Promoters

**Figure 5:**
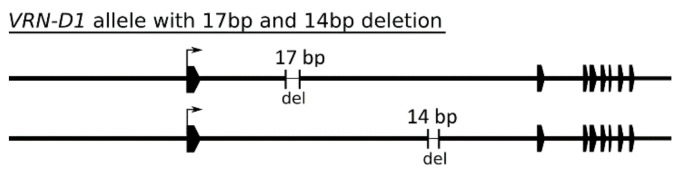
Schematic representation of *VRN*-*D1* alleles with 17-bp and 14-bp deletions within the first intron. Black polygons represent exons. The sizes of deletions are not to scale.

In the panel of 105 wheat cultivars, 96 possessed variants of the recessive *vrn*-*D1* allele. Six cultivars carried the dominant *Vrn*-*D1a* allele, one cultivar had the *Vrn*-*D1b* allele, and two cultivars had the *Vrn-D1c* allele. Thirteen cultivars carried 17-bp deletions and 10 cultivars carried 14-bp deletions in the first intron (Figure 5). These deletions were present in both dominant and recessive alleles. According to the sequence variability of the *VRN*-*D1* gene, the cultivars were divided into 9 groups (Supplementary Table S7). The largest group, Group 1D (64 cultivars), differs from Group 2D (including the reference TDC) by only an A/G SNP. Overall, the *VRN*-*D1* gene does not show sequence variability as high as that of its homoeologs.

The analysis of ≈1.2 kb of the promoter sequence (SM1) showed an insertion of 174 bp corresponding to the *Vrn*-*D1c* allele [16] in the cultivars Dalmatia 2 and 771-VII/12 and one SNP in the cultivar Botagoz distinguishing the *Vrn*-*D1b* allele from the *Vrn*-*D1a* allele [12,18]. Otherwise, no sequence polymorphisms were found (GenBank accession OK556478). All analysed cultivars shared 23 bp long and 19 bp long G4 motifs located near the VRN box (Supplementary Table S4).

#### 2.2.4. Comparison of *VRN1* Homoeologous Promoter Regions

In total, four G4 motifs were present within the 1 kb region of the *VRN*-*A1*, *VRN*-*B1* and *VRN*-*D1* promoters (Supplementary Table S4). A 23 bp long G4 was common for the *VRN*-*B1* and *VRN*-*D1* promoters (even though it differed in two SNPs) but disrupted in the *vrn*-*A1* promoter. This motif is positioned near two regulatory elements: VRN-box and CArG-box. Three other G4 motifs were unique to the promoters of *VRN*-*A1*, *VRN*-*B1* or *VRN*-*D1*. The longest G4 motif (34 bp) was observed in the *vrn*-*A1* promoter, 750 bp upstream of the start codon. A 32 bp G4 motif unique to *VRN*-*B1* occurs at a similar position (735 bp upstream of the start codon). Contrary to that of its homoeologs, shorter unique G4 of *VRN*-*D1* (19 bp) is situated only 310 bp upstream of the start site. Thus, both G4 motifs of *VRN*-*D1* are in proximity to regulatory regions containing VRN boxes and CArG boxes. In addition to the described G4 structure and numerous SNPs, several indels and polymorphic microsatellite loci distinguish the three recessive homoeologous promoters of the *VRN1* gene. Six microsatellite repeats were found within 1 kb of the *vrn*-*A1* and *VRN-B1* promoter sequences and three only in the *VRN*-*D1* promoter. As shown in Supplementary Table S8, three and two microsatellite repeats are unique to the *vrn*-*A1* and *VRN*-*B1* promoters, respectively. Conversely, all three microsatellite loci in the promoter of *VRN-D1* can also be found in those of its homoeologs.

### 2.3. Effect of VRN-A1 CNV on Heading Time

**Figure 6:**
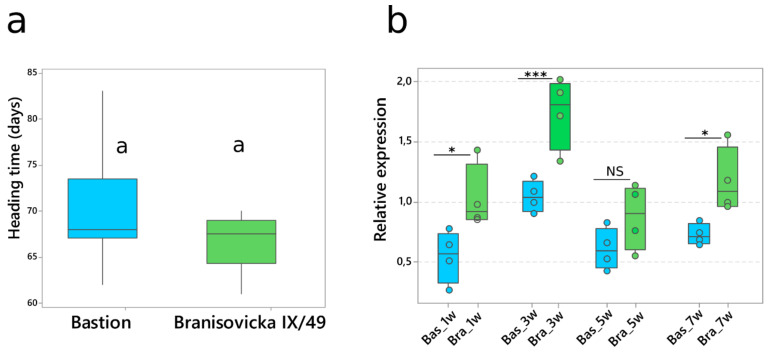
The impact of *Vrn*-*A1a* CNV on heading time and gene expression. (**a**) Box plot of mean heading times (twelve plants of each variety) for Bastion and Branisovicka IX/49 carrying one and two *Vrn*-*A1a* copies, respectively. Means that do not share the same letter are significantly different according to Tukey’s test (*p* < 0.05). (**b**) Time-course expression analysis of one and two copies of *Vrn*-*A1a*. Plants were sampled at weeks one, three, five and seven. Circles represent individual data points from four biological replicates. Each replicate represents a pool of three plants. Bas—Bastion (one copy), Bra—Branisovicka IX/49 (two copies). * *p* value < 0.05, *** *p* value < 0.001, NS—not significant. Significance determined by Student’s *t*-test.

The identification of the same allelic composition (*Vrn*-*A1a*, *Vrn*-*B1c*, *vrn*-*D1*, *Ppd*-*A1a*, *Ppd*-*B1b* and *Ppd*-*D1b*) but different numbers of *Vrn*-*A1a* copies (one copy in Bastion and two copies in Branisovicka IX/49) in the spring wheat cultivars Bastion and Branisovicka IX/49 provided an opportunity to assess the impact of CNV on heading time. The mean heading times were 66.7 and 69.7 days for Branisovicka IX/49 and Bastion, respectively (Figure 6a), and the difference was not statistically significant. The *Vrn*-*A1a* expression level was significantly higher in Branisovicka IX/49 than in Bastion at weeks one, three and seven. Surprisingly, the expression level decreased at week five to the level observed at week one in both varieties. The transcription level in Branisovicka IX/49 increased again at week seven but did not reach the level observed at the third week (Figure 6b).

## 3. Discussion

Winter wheat plants have an intact *VRN1* gene, while spring wheat plants carry mutations in the promoter or the first intron, affecting the regulatory regions. Better knowledge of *VRN1* sequence variation may improve the understanding of the vernalization mechanism. We sequenced a panel of 105 hexaploid wheat cultivars, including both winter and spring cultivars with different countries of origin, to cover the broad spectrum of possible allelic variants, and sequenced their *VRN1* genes.

### 3.1. VRN1 Sequence Variability

Generally, the *VRN1* gene showed high sequence similarity across the allelic variants of each homoeolog in our study. The most variable gene was *VRN-A1*. According to the *vrn*-*A1* nucleotide sequence pattern, 105 cultivars were divided into 20 groups (Supplementary Table S2). Illumina data also provide insight into the sequence variability between *vrn*-*A1* copies. Two or more copies of the recessive *vrn*-*A1* allele in hexaploid wheat were reported to be associated with the C/T SNP in exon 4 (Ex4C/T) and the T variant in exon 7 (Ex7T) [4]. It was suggested that the exon 7 polymorphism originated in a wild tetraploid species (*Triticum diccocoides* Körn), while the mutation in exon 4 originated later in hexaploid wheat [38]. Cultivars forming Group 8 carry two copies of *vrn*-*A1*, but they do not show the Ex4C/T variants or any other SNPs at the same nucleotide position, indicating the presence of two different copies. On the other hand, they do carry Ex7T. Otherwise, the presence of multiple copies of *vrn*-*A1* in 65 of 70 cultivars was associated with the Ex4C/4T/7T haplotype. The *Vrn*-*A1a* and *Vrn*-*A1B* dominant alleles present in two copies carry an intact Ex4C/7C haplotype, supporting the observation of Muterko and Salina [38].

In the present study, we revealed that the *Vrn*-*A1B* allele, carrying mutations in the promoter region [15], also contains a 177 bp insertion in the first intron. The insertion was found within the “critical region” of intron 1 near the putative regulatory RIP3 site in spring cultivars carrying the *Vrn*-*A1B* allele. The same insertion was also detected in several tetraploid cultivars possessing the *Vrn*-*A1B* allele (unpublished data). The influence of two different mutations located in the promoter and intron 1 on the expression of *Vrn*-*A1B* remains unclear. We also confirmed the presence of the 177 bp insertion in one of the *vrn*-*A1* alternative splice variants. To date, two alternative splice variants have been described: the full *vrn*-*A1* transcript corresponding to the complete gene (later designated *VRNA1*-long) and a 600 bp long alternative splice variant designated *VRNA1*-short [13,14]. Sequencing of the *VRNA*1-short transcripts of cultivars with *Vrn*-*A1B* revealed two variants, the *VRNA*1-short transcript with the 177 bp insertion and the *VRNA*1-short transcript without the insertion, which contains a number of SNPs in comparison with the *Vrn*-*A1B* genomic sequence. The *Vrn*-*A1b* allele is not always associated with spring growth habits in tetraploid and hexaploid wheat lines [39,40]. We supported this observation by screening a set of 95 wild emmer wheat cultivars, where the *Vrn*-*A1b* allele (including the 177 bp insertion) was detected in both spring and winter cultivars (data not shown). The influence of *Vrn*-*A1b* on the spring habit of Pyrothrix 28, Rescue and VL-30 cannot be precisely determined due to the presence of other dominant alleles (*Vrn*-*B1c*, *Vrn*-*B1a* and *Vrn*-*D4*, respectively) in these cultivars.

Regarding sequence polymorphism, the *VRN*-*B1* gene was divided into 20 groups, among which Groups 1B–15B comprise recessive *vrn*-*B1* haplotypes (Supplementary Table S5). The novel allele (forming Group 20B) designated *Vrn*-*B1f* displayed an interesting expression profile. The comparison of two dominant alleles, *Vrn*-*B1a* and *Vrn*-*B1c*, showed that *Vrn*-*B1a* in the cultivar Baroota 8791 (mean heading time of 93 days) had the lowest expression. The basal expression level of *Vrn*-*B1c* (cultivar Paragon) was significantly higher than that of the *Vrn*-*B1a* and *Vrn*-*B1f* alleles (cultivar Barta) and gradually increased over time. One should not overlook the possibility the higher expression level of *Vrn*-*B1c* and *Vrn*-*B1f* alleles could be also promoted by the presence of dominant *Vrn*-*A1a* allele. Loukoianov et al. [41] suggested expression of dominant *Vrn*-1 alleles can positively affect the expression of recessive *vrn*-1 alleles. The mean heading time was similar between Barta (57.5 days) and Paragon (59.5 days), which could be explained by a sudden increase in *Vrn*-*B1f* expression between the third and fifth weeks (Figure 4). Emtseva et al. [42] reported that non-vernalized plants with *Vrn*-*B1c* headed earlier than plants with *Vrn*-*B1a*, which is consistent with our observation. On the other hand, vernalization leads to greater heading time acceleration in lines carrying weaker *Vrn*-*B1a* alleles [42]. Our results suggest that the strength of the new *Vrn*-*B1f* allele is similar to that of *Vrn-B1c*, yet the mechanism of regulation most likely differs. 

The *VRN*-*D1* gene and its promoter are highly conserved. Contrary to its homoeologs, both of which formed 20 haplotype groups, *VRN*-*D1* formed only 9 groups according to its detected sequence variability among 105 cultivars (Supplementary Table S7). In addition to known mutations of the *Vrn*-*D1a*, *Vrn*-*D1b* and *Vrn*-*D1c* alleles, only several SNPs and abundant 14 bp and 17 bp deletions in the first intron were found.

### 3.2. VRN1 Promoter Secondary Structures

The formation of four-stranded nucleic acid structures may affect the accessibility of genomic regions [43]. Muterko et al. [44] hypothesized that the *C*-rich region of the VRN box regulates *VRN1* transcription through the formation of quadruplex structures that are destabilized in the dominant *Vrn*-*A1b* or *Vrn*-*Am1a* allele due to the presence of SNPs and deletions, respectively. It was noted that G-quadruplexes may also cause sequencing errors [37]. We detected a 34 bp long G-quadruplex located 784 bp upstream of the start codon in the *vrn*-*A1* promoter of TDC (MH347747) after encountering problems with amplification and Sanger sequencing of part of the *vrn*-*A1* promoter. Only sequences of promoter variants with deletions spanning the G4 region were obtained. In addition to the longest G4 motif unique to the *vrn*-*A1* promoter, one shared G4 and one unique G4 were found in each of the *VRN*-*B1* and *VRN*-*D1* promoters. Overall, over one million G4 motifs preferentially located within 500–1000 bp upstream of the start codon were identified in the bread wheat genome [45].

A growing number of results suggest the involvement of G4s in transcription regulation and were recently suggested as new epigenetic regulators of transcription [46,47]. In fact, G4s are prevalent TF binding sites in human chromatin, promoting increased transcription [48]. In plants, knowledge of G4 function remains limited. A genome-wide analysis of G4s revealed sequence and functional conservation among monocots, and several G4-containing genes were found to be conserved between wheat and barley [49]. Our results indicate a role of G4 motifs in the co-regulation of *VRN1* homoeologs. Deletions spanning G4 motifs in the promoters of recessive *vrn*-*A1* alleles of winter cultivars with more than one gene copy (Figure 3) might explain why the increased number of recessive *vrn*-*A1* gene copies does not accelerate flowering. We hypothesize that *vrn*-*A1* copies with a disrupted G4 may not be transcriptionally activated during vernalization, but they may still bind potential transcriptional activators, serving as a decoy, and modulate the expression of active copies. This could lead to a prolonged vernalization requirement in cultivars with more copies of recessive *vrn*-*A1*, as observed by Díaz et al. [4]. It should be noted that Li et al. [31] proposed that early flowering of winter wheat Jagger and late flowering of winter wheat 2174 after three-week vernalization was caused by different amino acid residues in the *C*-terminal region of VRN1. Jagger carries one copy of *vrn*-*A1a* allele (Ala^180^) while 2174 carries two copies of *Vrn*-*A1B* allele (Val^180^). Later, identification of two RIP3 haplotypes in the same winter wheat cultivars (Jagger 3_SNPs and 2174 1_SNP) [14] complicated the interpretation of obtained results. Further investigation using population segregating for individual G4s or CRISPR/Cas9-edited lines would help to understand the effect of G4 on flowering time.

### 3.3. VRN1 Copy Number Variation

CNV at the *vrn*-*A1* locus was reported by Díaz et al. [4], who linked the previously described C/T SNP in exon 4 [50] with the presence of other *vrn*-*A1* copies. Plants with more *vrn*-*A1* copies required longer cold exposure for the transition from the vegetative to reproductive stage [4]. Wheat varieties with a higher number of *vrn*-*A1* copies are grown in countries with a more continental climate, which suggests that *vrn*-*A1* CNV plays a role in wheat adaptation to different climates [29]. The CNV and haplotype of *vrn*-*A1* were also found to be associated with frost tolerance in bread wheat [51]. More recently, a duplication of *vrn*-*A1* alleles was observed in hexaploid wheat, as well as duplication of *VRN*-*B1* in *Triticum compactum* (Host) and *T. spelta* (L.) [27]. In this work, CNV of *VRN*-*A1*, *VRN*-*B1* and *VRN*-*D1* was determined in a panel of 105 hexaploid cultivars. While the maximum copy number of the recessive allele *vrn*-*A1* was four, dominant *vrn*-*A1* alleles were present in only one or two copies.

Heading time and *Vrn*-*A1a* expression analyses of Bastion with one copy of *Vrn*-*A1a* and Branisovicka IX/49 with two copies yielded surprising results. We expected Bastion to head significantly later, as the presence of two copies of *Vrn*-*A1a* in the Branisovicka IX/49 cultivar should result in a higher level of transcription and hence more VRN-A1 protein, resulting in earlier heading. Although the transcription levels of the two copies were nearly two-fold higher, the impact on heading time was not significant. More interestingly, the sudden decrease in week five may represent a certain mechanism of self-regulation. A similar observation was made by Loukoianov et al. [41]. The initial transcription level of *Vrn*-*A1a* at the first leaf stage was low, after which the level increased at the second leaf stage but decreased at the third leaf stage. Additionally, we did not find more than two copies of the dominant *Vrn*-*A1a* allele among the varieties we examined. Although Bastion and Branisovicka IX/49 carry the same *VRN1* and *PPD1* alleles, one cannot rule out the influence of different genetic background on heading time. 

The *Vrn*-*A1a* allele is considered the strongest and the most actively transcribed dominant *VRN1* allele [41,52]. We can speculate that both findings—the sudden decrease in *Vrn*-*A1a* expression during plant development and the maximum of two copies of *Vrn*-*A1a*—indicate that some sort of two-level (fast and slow) self-regulatory mechanism is involved. Fast regulation can be observed at the transcriptional level and could be explained by mRNA degradation [53]. Slow regulation resulting in a maximum of two *Vrn*-*A1a* copies may be an evolutionary adaptation, as suggested in the model by Jędrak et al. [54]. Dosage-reversed CNVs were reported, for instance, in *Drosophila melanogaster*, where 8% of CNVs showed a negative association between gene expression and the copy number of genes [55], and in the human genome. This may indicate regulation by dosage compensation mechanisms, such as mRNA degradation [56]. In plants, similar observations were recently made. In *Oryza sativa*, 4.5% of analyzed genes showed a negative correlation, 82.32% showed no significant correlations, and only 13.7% of genes showed a significant positive correlation with copy number [57].

In allopolyploid bread wheat, homoeolog-specific gene transcription can be balanced and/or accompanied by massive homoeolog-specific up- and downregulation of gene expression [58]. Ramírez-González et al. [59] showed that approximately 30% of wheat homoeolog triads (A, B and D) have an unbalanced expression pattern, with higher or lower expression from one of the homoeologs. Similarly, vernalized winter TDC shows a higher expression level of *vrn*-*A1* than of *vrn*-*B1* and *vrn*-*D1* [41]. Our in-depth genomic sequence analysis of *VRN1* triads, including promoter sequences, revealed higher variability within the *vrn*-*A1* homoeolog. Notably, in our set of 105 wheat cultivars, more copies of the *VRN1* gene were detected exclusively in the A subgenome. *VRN1* gene function has been linked with the transition from the vegetative to the reproductive stage. A recent study suggested association of root phenotype with the *VRN1* allelic variant in wheat and barley [60]. Wheat varieties carrying the recessive *vrn*-*B1* allele had a significantly narrower seminal root angle than varieties with the dominant *Vrn*-*B1* allele. This surprising pleiotropic effect suggests that analysis of the expression profiles of all three homoeologs and their allelic variants in different vernalized and non-vernalized tissues may shed light on homoeolog-specific gene transcription.

Our results add another piece into the jigsaw puzzle called vernalization, as they: provide complete sequences of *VRN1* homoeologs; describe the structure of the novel allele, including its expression profile; suggest a putative role of the G4 secondary structures within the promoter sequence in *VRN1* transcription; and reveal the possible impact of *Vrn*-*A1a* CNV on wheat heading time.

## 4. Materials and Methods

### 4.1. Plant Material

A total of 105 bread wheat (*T. aestivum* L.) cultivars, comprising 65 winter and 40 spring cultivars with diverse geographical origins, were used in this study (Supplementary Table S1 and Figure S6). Seeds of all but three cultivars were provided by the Crop Research Institute Gene Bank (Prague, Czech Republic). Winter wheat cultivars Jagger and Elly were kindly provided by Eduard Akhunov (Kansas State University, Manhattan, KS, USA) and Tibor Sedláček (Selgen, Czech Republic), respectively, while seeds of the near-isogenic line Triple Dirk C (TDC) were kindly provided by Jorge Dubcovsky (Davis, UC, USA).

### 4.2. DNA Extraction and Genotyping

Genomic DNA was extracted from the leaves of two-week-old plants using a NucleoSpin Plant II Kit (MACHEREY-NAGEL, Dueren, Germany) according to the manufacturer´s instructions. DNA amplification was performed using a C1000 Touch Thermal Cycler (Bio-Rad, Hercules, CA, USA) with the primers and PCR conditions listed in Supplementary Table S9. Primers reported by Fu et al., Yan et al. and Milec et al. [12,15,19] were used for *VRN1* genotyping. The *Ppd*-*A1* allele was determined following [61], and the *Ppd*-*D1* allele was determined following [2]. New primers for *VRN1* sequencing were designed using Primer3 2.3.7 [62] as part of Geneious Prime^®^ 2021.2.2 (https://www.geneious.com). To sequence all three homoeologous *VRN1* loci, several overlapping regions were amplified (Supplementary Figure S7). Long amplicons (from 6 to 11 kb) were amplified by PrimeSTAR GXL DNA Polymerase (Takara Bio, Kusatsu, Japan) and Expand Long Range, dNTPack (Roche, Basel, Switzerland), and short amplicons (from 600 bp to 3 kb) were amplified by HOT FIREPol DNA Polymerase (Solis BioDyne, Tartu, Estonia), all according to the manufacturer’s instructions. The specificity of all primer pairs was tested on DNA from nulli-tetrasomic lines of cv. Chinese Spring (N5AT5B, N5AT5D, N5BT5A, N5BT5D, N5DT5A and N5DT5B).

### 4.3. A Chromosome Sorting by Flow Cytometry

Suspensions of intact mitotic metaphase chromosomes were prepared from synchronized root tips of young seedlings of bread wheat (*T. aestivum* L.) as described in [63], including labelling with an Alexa488-tagged GAA7 probe following [64]. Chromosome samples were stained with DAPI at a final concentration of 2 µg/mL and analyzed at a rate of 2000 chromosomes per second on a BD FACSAria SORP flow cytometer and sorter (BD Biosciences, San Jose, CA, USA). Initial gating was performed with a forward scatter vs. DAPI scatter plot, and a subsequent dependent sorting gate for chromosome 5A was drawn as a DAPI vs. FITC bivariate scatter plot (Supplementary Figure S8). In total, 20,000 to 80,000 chromosomes per cultivar were sorted in 40 µL of ddH_2_O.

### 4.4. CNV of VRN1 Homoeologs and Ppd-B1

Determination of *vrn*-*A1* copies in the complete panel of 105 cultivars and *Ppd*-*B1* copies in eight spring cultivars selected from this panel was performed by iDna Genetics (Norwich, UK) using the TaqMan^®^ CNV assay [4]. The estimation of *VRN*-*B1* and *VRN*-*D1* copies was performed by digital droplet PCR (ddPCR) in house. Prior to ddPCR, DNA was digested with the restriction enzyme *Hin*dIII-HF (cat. R3104S, New England Biolabs, Ipswich, MA, USA) according to the manufacturer’s instructions. For each sample, 800 ng of genomic DNA was used for digestion. ddPCR analysis was performed using ddPCR™ Supermix for Probes (no dUTP) (Bio-Rad, Hercules, CA, USA) according to the manufacturer’s instructions with a 60 °C annealing/extension phase and 40 ng of digested DNA for each sample. The copy number of *VRN*-*B1* was determined using primers and a TaqMan^®^ probe (Thermo Fisher Scientific, Waltham, MA, USA) as described by Guedira et al. [28]. For *VRN*-*D1* copy number estimates, we designed primers and a TaqMan^®^ probe localized to exon 2. The specificity of the *VRN*-*D1* TaqMan^®^ probe was validated using nullisomic-tetrasomic lines (N5AT5D, N5BT5A and N5DT5A). All primers and TaqMan^®^ probes are listed in Table 1.

### 4.5. Sequencing of VRN1 Homoeologs

The length of the *VRN1* gene and its allelic variants, together with CNV of *vrn*-*A1* and similarity of A, B and D homoeologs, hampered the acquisition of desired amplicons for sequencing and sequence data analysis. To ensure PCR product specificity, different approaches were implemented, and both genomic DNA and DNA from flow-sorted chromosome 5A were used for PCR. To correctly interpret the obtained sequence data, CNV of *VRN*-*A1*, *VRN*-*B1* and *VRN*-*D1* genes was estimated before sequencing. Sequences of *VRN1* genes and their upstream regions were obtained after sequencing overlapping PCR products using three protocols. Short PCR products (<1200 bp) were sequenced by the Sanger method, while long PCR products (>2700 bp) were sequenced on the Illumina iSeq platform (Supplementary Figure S9). The PCR amplicon (primers vrn-B1_4F/4R) from cv. Anza showed a duplicated inserted sequence; therefore, it was resequenced using the Oxford Nanopore Technologies approach. As attempts to design specific primers amplifying the *vrn*-*A1* promoter for Illumina sequencing failed, a set of published primer pairs was used to amplify and Sanger sequence the promoter sequence [14]. PCR of DNA from nulli-tetrasomic lines of Chinese Spring (N5AT5B, N5AT5D, N5BT5A, N5BT5D, N5DT5A and N5DT5B) revealed that the three primer pairs were not specific to the *vrn*-*A1* gene. To ensure amplification specificity, we used DNA of flow-sorted 5A chromosomes of 29 selected cultivars (Supplementary Table S1) for *vrn*-*A1* promoter sequencing. Eventually, to analyze important regulatory promoter regions containing VRN boxes and CArG boxes in all 105 cultivars, the previously published primer pair VRN1AF/VRN1-INT1R [15] was used.

#### 4.5.1. Sanger Sequencing

PCR clean-up was performed by ExoSap (Thermo Fisher Scientific, Waltham, MA, USA). The sequencing reactions were performed using the BigDye1 Terminator v.3.1 Cycle Sequencing Kit (Applied Biosystems, Waltham, MA, USA) and purified using the Agencourt Clean SEQ Dye-Terminator Removal Kit (Beckmann Coulter, Brea, CA, USA). The reactions were analyzed on an ABI3730xl DNA analyzer (Applied Biosystems, Waltham, MA, USA). The sequences were trimmed and assembled using Geneious Prime^®^ 2021.2.2 (http://www.geneious.com). The assemblies were verified by alignment with the reference sequences of TDC (AY747600.1, AY747604.1 and AY747606.1).

#### 4.5.2. Illumina Sequencing

PCR amplicons were purified using AMPure XP Beads (Beckman Coulter, Brea, CA, USA) with a DNA volume/beads ratio of 1:1. DNA was quantified using the Qubit dsDNA HS assay system (Invitrogen, Waltham, MA, USA). For each PCR amplicon or pool of amplicons, a sequencing library was prepared using the NEBNext^®^ Ultra™ II DNA Library Prep Kit for Illumina^®^ with the following modifications: (i) DNA was fragmented in 50 µL solution using a Bioruptor Plus (Diagenode, Liège, Belgium) eight times for 30 s on the HIGH setting; (ii) size selection was performed for an approximate insert size of 500–700 bp; and (iii) PCR enrichment was carried out in 3–4 cycles. Libraries were equimolarly pooled and sequenced on an Illumina iSeq system with 150 bp paired-end (PE) reads to achieve a minimal amplicon coverage of 100×.

#### 4.5.3. Oxford Nanopore Sequencing

A sequencing library was prepared using a Ligation 1D Kit SQK-LSK109 (Oxford Nanopore Technologies, Oxford, UK) according to the protocol provided by Oxford Nanopore Technologies (ONT). The completed library was loaded into a Nanopore MinION Spot-ON Flow Cell (FLO-MIN106D, v.R9) and sequenced. Data were collected for 36 h, and base calling of the raw data was performed using Guppy (v. 4.2.2). The ONT reads were de novo assembled by Flye (v. 2.8).

#### 4.5.4. Different Variants of the *vrn*-*A1* Promoter

*VRN*-*A1* promoter amplicons (primers VRN1_prom_F3/R3 and VRN1_prom_F4/R5) were cloned prior to Sanger sequencing using the CloneJET PCR Cloning Kit (Thermo Fisher Scientific, Waltham, MA, USA).

### 4.6. Sequencing Data Analysis

The isogenic line TDC with intact *VRN1* alleles was set as the reference sequence. *VRN1* genes were resequenced using the primers listed in Supplementary Table S9, and the resulting sequences were compared with previously published sequences [12]. The sequence published by Kippes et al. [14] was used as the reference sequence for the *vrn*-*A1* promoter. The remaining *vrn*-*B1* and *vrn*-*D1* upstream region reference sequences were obtained by designing new primers (Supplementary Table S9) using sequences of cv. Chinese Spring available from Ensembl Plants (http://plants.ensembl.org/index.html, accessed on 10 February 2020). DNA from TDC was used as a template for PCR, and the resultant PCR products were sequenced on the Illumina iSeq platform. The sequence data obtained were analyzed as described below, and trimmed reads were mapped to the sequences from Ensembl Plants. The sequences from TDC were subsequently used as reference sequences to map short Illumina reads.

Read trimming based on quality (Q30) and sequencing adaptor removal were performed with Trimmomatic (v.0.32) [65]. All trimmed reads for each sample were mapped to the *VRN1* TDC reference with BWA-MEM (v.0.7.15) [66]. Mapped reads for each genome variant (A, B and D) were extracted from the bam file by SAMtools (v.1.9) [67] and de novo assembled by Spades (v.3.13.0) [68]. Mapping results were manually reviewed with Integrative Genome Viewer v.2.6.3 (IGV) [69], and the sequences were further analyzed in Geneious Prime^®^ 2021.2.2 (http://www.geneious.com).

Final sequences of different lengths were obtained for the *vrn*-*A1* (300 bp for all 105 cultivars when using the VRN1AF/VRN1-INT1R primer pair [15] and 2.2 kb for 29 selected cultivars when using DNA from flow-sorted 5A chromosomes and the primer set designed by [14], excluding the 300 bp amplified with the VRN1_prom_F3/VRN1_prom_R3 primers), *VRN-B1* (4.5 kb) and *VRN*-*D1* (1.2 kb) promoters of 105 sequenced cultivars. Due to the overall high sequence homology, only a 1 kb portion of the homoeologous *VRN1* promoters of the sequenced representative cultivar TDC was chosen for the comparative analysis.

Prediction of non-canonical DNA structure conformations was performed using the GrainGenes database (https://wheat.pw.usda.gov/GG3, accessed on 20 July 2021) [70], DNA fold prediction of G4 motif was performed by the Vienna package RNAfold tool as part of Geneious Prime^®^ 2021.2.2 (https://www.geneious.com), and microsatellite analysis was performed using the online tool Microsatellite repeats finder [71], available at http://insilico.ehu.es/mini_tools/microsatellites/ (accessed on 22 July 2021). New allelic sequences are deposited in NCBI database (GenBank accessions MZ593843, MZ593844, MZ593845, OK556477 and OK556478).

### 4.7. Growth Conditions

Heading time experiments were performed with two spring wheat varieties, Bastion and Branisovicka IX/49, differing in the number of *Vrn*-*A1a* copies. Seeds were imbibed in Petri dishes at 22 °C for 24 h and then kept at 4 °C for two days to synchronize germination. Twelve seedlings of each variety were transferred to pots and placed in a growth chamber set to long-day conditions (16 h of light at 20 °C and 8 h of darkness at 16 °C). The heading date was recorded when half of the first spike had emerged. For gene expression analysis, leaves were collected one week, three weeks, five weeks and seven weeks after potting. After we identified *VRN1* sequence variants in other cultivars, we also performed heading time and expression studies. The same conditions as described above were used to grow the winter line TDC and selected spring varieties: Barta, Baroota 8791, and Paragon. Plants were grown for RNA extraction under the same conditions, and samples were collected from 1-week-old, 3-week-old and 5-week-old plants. We also analyzed the expression of *vrn*-*A1* alternative splice variants revealed in this study. The last expanded leaf was sampled at weeks three, four and five from the winter line TDC (one copy of *vrn*-*A1*), the spring cultivars Rescue (two copies of *Vrn*-*A1b*) and VL-30 (two copies of *Vrn*-*A1B* and 1 copy of *Vrn*-*D4*).

### 4.8. RNA Extraction and Gene Expression

Total RNA was extracted from leaves using a RNeasy Mini Kit (Qiagen, Hilden, Germany) according to the manufacturer’s instructions. cDNA was synthesized using a Transcriptor High Fidelity cDNA Synthesis Kit (Roche, Basel, Switzerland) according to the manufacturer’s instructions with 2 μg of total RNA and anchored-oligo (dT)18 primers. DNA was removed during RNA purification using the RNase-Free DNase Set (Qiagen, Hilden, Germany). The gene expression level was determined using reverse transcription-qPCR (RT–qPCR). RT–qPCR was performed using 2 × SYBR Master Mix (Top-Bio, Prague, Czech Republic) on the CFX96^TM^ Real-Time PCR Detection System (Bio–Rad, Hercules, CA, USA). The data were analyzed using the 2^−ΔΔCq^ method with CFX Maestro 2.0 software (Bio–Rad, Hercules, CA, USA). Three replicate PCR amplifications were performed for each sample. The expression level was standardized against the reference glyceraldehyde-3-phosphate dehydrogenase (*GAPDH*) according to Ivaničová et al. [72]. New primers for detecting *VRN*-*B1* expression level were designed in this study. The sequences of all primers used for RT–qPCR are listed in Supplementary Table S6. Gene expression studies were performed using at least three biological replicates.

## 5. Conclusions

*VRN1* is main vernalization gene in wheat. Here we report in-depth sequence analysis of complete *VRN1* homoeologs (A, B and D) including their promoter regions in the panel of 105 winter and spring varieties of hexaploid wheat for the first time. Copy number variation analysis of *VRN1* homoeologs showed that *VRN*-*B1* and *VRN*-*D1* are present in only one copy in comparison to recessive *vrn*-*A1*, which ranged from one to four copies. An integral part of the results is development of original methodology for sequencing of the complete *VRN1* genes (13 kb). We have also introduced the method for determination of *VRN*-*B1* and *VRN*-*D1* copy number variation by droplet digital PCR.

## Figures and Tables

**Table 1:** Sequences of primers and probes used for the determination of *VRN-B1* and *VRN*-*D1* copy number variation using ddPCR assay. The TaqMan^®^ (taq) probes were labelled with either FAM (5B chromosome, 5D, target) or VIC (3D chromosome, reference).

Oligo ID	5′–3′ Sequence and Modifications	Amplicon Length	Reference
Ta-3D_F	CTCATCTCAGGCTGTCTAATTAA	167 bp	[36]
Ta-3D_R	CATAGATCCCTCCTTGAAGGA		
Ta-3D_taq	VIC-CCTCACTCAAGCACCACATCG-QSY		
CNV_VRNB1_F	CAGCATTCATCCAGCGGCAT	114 bp	[28]
CNV_VRNB1_R	CTTCAGCCGTTGATGTGGCTA		
CNV_VRNB1_taq	FAM-CAGAGGATGCGGCAGTGCAG-QSY		
CNV_VRND1_F	AAATTCTTGAACGGTATGAGCGCTAC	109 bp	This study
CNV_VRND1_R	GCTAAAGGAAAGCAAACCATTTG		
CNV_VRND1_taq	FAM-TGCAGAAAAGGTTCTCGTTTCAAGTG-QSY		

**Table 2:** Copy number variation (CNV) of recessive and dominant *VRN1* alleles in 105 hexaploid wheat cultivars. The values indicate the number of varieties carrying the respective CNV. Three copies of *vrn*-*A1* were detected in cultivar VL-30, but one of them was proven the *Vrn*-*D4* allele.

CNV	Winter Wheats			Spring Wheats					
	*vrn*-*A1*	*vrn*-*B1*	*vrn*-*D1*	*vrn*-*A1*	*Vrn*-*A1a*	*Vrn*-*A1B*	*VRN*-*B1*	*VRN*-*D1*	*Vrn*-*D4*
1	4	65	65	4	15	-	40	40	1
2	18	-	-	6	8	3	-	-	-
3	41	-	-	4	-	-	-	-	-
4	2	-	-	-	-	-	-	-	-

## Data Availability

All data generated or analyzed during this study are included in this published article and its supplementary information files. New allelic sequences are deposited in NCBI database.

## Supplementary Materials

The following are available online at https://www.mdpi.com/article/10.3390/ijms222212284/s1. ESM1 (.zip): FASTA alignments (*. fasta); ESM2 (.xlsx): Table S1: List of *Triticum aestivum* cultivars, their growth habit, country of origin, CNV of *VRN*-*A1* and allelic composition, Table S2: The 105 wheat cultivars grouped according the sequence variability of *VRN*-*A1* gene body, Table S3: Primers used to detect allelic variants of *VRN*-1, Table S4: G4 motifs in *VRN*-1 homoeologous promoters, Table S5: The 105 wheat cultivars grouped according the sequence variability of *VRN*-*B1* gene body, Table S6: Primers used for the RT-qPCR, Table S7: The 105 wheat cultivars grouped according the sequence variability of *VRN*-*D1* gene body, Table S8: Shared microsatellite tandem repeats between homoeologs are highlighted with the same color, Table S9: PCR conditions and sequencing platform used for *VRN*-1 and *VRN*-1 upstream region sequencing; ESM3 (.PDF): Figure S1: Agarose gel electrophoresis (1,5% agarose) of PCR products distiguishing recessive *vrn*-*A1* allele and dominant *Vrn*-*A1b* allele with 177 bp insertion, Figure S2: Agarose gel electrophoresis (1,2% agarose) of RT-PCR products, Figure S3: Alignment of *VRNA1*-short transcript variants, Figure S4: Agarose gel electrophoresis (0,8% agarose) of vrnB1_4F/R PCR products amplifying *VRN*-*B1* with and without 837 bp insertion, Figure S5: Standard curve of q.VRNB1 for 2 fold dilution series, Figure S6: Geographical origin of 105 hexaploid wheat cultivars, Figure S7: Overlapping amplicons used for *VRN*-*A1*, *VRN*-*B1* and *VRN*-*D1* sequencing, Figure S8: Flow sorting of chromosome 5A, Figure S9: Schematic representation of recessive and dominant *VRN*-1 alleles and their regions sequenced by different sequencing platforms.
