# Supplementary material for: In-Depth Sequence Analysis of Bread Wheat VRN1 Genes
Source: Int J Mol Sci. 2021 Nov 13;22(22):12284. doi: 10.3390/ijms222212284 (PMC8626038; doi:10.3390/ijms222212284)
Supplement: Supplementary file 1 [file ijms-22-12284-s001.zip › ESM3.pdf]

SUPPLEMENTARY INFORMATION

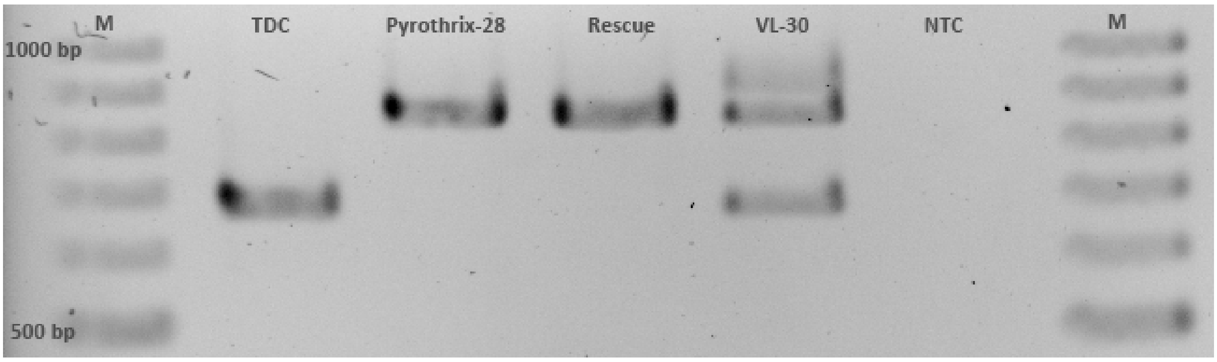

Figure S1: Agarose gel electrophoresis (1.5% agarose) of PCR products distiguishing recessive *vrn-A1* allele and dominant *Vrn-A1b* allele with 177bp insertion. TDC – 677 bp (*vrn-A1*), Pyrothrix-28 and Rescue – 854 bp (*Vrn-A1b*), VL-30 – 677 bp (*Vrn-D4*) and 854 bp (*Vrn-A1b*), NTC (no template control) – no product. Lane M: GeneRuler 100 bp Plus DNA Ladder.

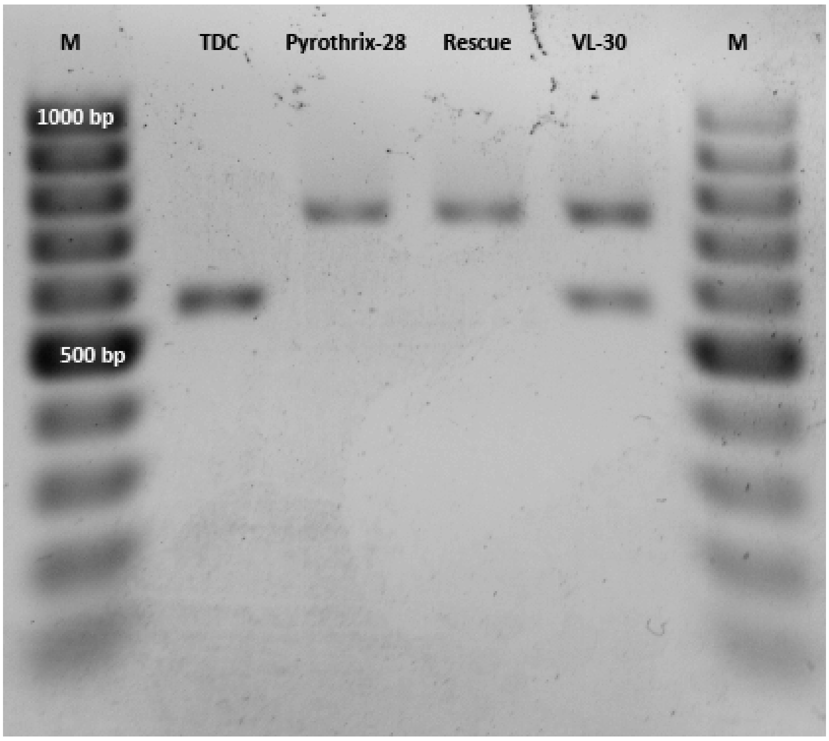

Figure S2: Agarose gel electrophoresis (1.2% agarose) of RT-PCR products amplified with VRNA1S_177inF/R primers distiguishing *VRNA1*-short transcript variant with and without 177bp insertion. TDC – 574 bp (*vrn-A1*), Pyrothrix-28 and Rescue – 751 bp (*Vrn-A1b*), VL-30 – 574 bp (*Vrn-D4*) and 751 bp (*Vrn-A1b*). Lane M: GeneRuler 100 bp DNA Ladder.

```
VRN-A1s            TTTTGGCCTGGCCATCCTCCCTCTCCTCCCCTCTCTTCCACCTCACGTCCTCACCCAACC   60
VRNA1-short_177in  --------------------------------------------------TCACCCAACC   10
VRNA1-short_SNPs   --------------------------------------------------TCACCCAACC   10

VRN-A1s            ACCTGATAGCCATGGCTCCGCCGCCTCGCCTCCGCCTGCGCCAGTCGGAGTAGCCGTCGC  120
VRNA1-short_177in  ACCTGATAGCCATGGCTCCGCCGCCTCGCCTCCGCCTGCGCCAGTCGGAGTAGCCGTCGC   70
VRNA1-short_SNPs   ACCTGATAGCCATGGCTCCGCCCCCCCGCCCCCGCCTGCGCCTGTCGGAGTAGCCGTCGC   70

VRN-A1s            GGTCTGCCGGTGTTGGAGGGTAGGGGCGTAGGGTTGGCCCGGTTCTCGAGCGGAGATGGG  180
VRNA1-short_177in  GGTCTGCCGGTGTTGGAGGGTAGGGGCGTAGGGTTGGCCCGGTTCTCGAGCGGAGATGGG  130
VRNA1-short_SNPs   GGTCTGCCGGTGTTGGAGGCTTGGGGTGTAGGGTTGGCCCCGTTCTCCAGCGGAGATGGG  130

VRN-A1s            GCGGGGGAAGGTGCAGCTGAAGCGGATCGAGAACAAGATCAACCGGCAGGTGACCTTCTC  240
VRNA1-short_177in  GCGGGGGAAGGTGCAGCTGAAGCGGATCGAGAACAAGATCAACCGGCAGGTGACCTTCTC  190
VRNA1-short_SNPs   GCGCGGGAAGGTGCAGCTGAAGCGGATCGAGAACAAGATCAACCGGCAGGTGACCTTCTC  190

VRN-A1s            CAAGCGCCGCTCGGGGCTTCTCAAGAAGGCGCACGAGATCTCCGTGCTCTGCGACGCCGA  300
VRNA1-short_177in  CAAGCGCCGCTCGGGGCTTCTCAAGAAGGCGCACGAGATCTCCGTGCTCTGCGACGCCGA  250
VRNA1-short_SNPs   CAAGCGCCGCTCGGGGCTGCTCAAGAAGGCGCACGAGATCTCCGTGCTCTGCGACGCCGA  250

VRN-A1s            GGTCGGCCTCATCATCTTCTCCACCAAGGGAAAGCTCTACGAGTTCTCCACCGAGTCATG  360
VRNA1-short_177in  GGTCGGCCTCATCATCTTCTCCACCAAGGGAAAGCTCTACGAGTTCTCCACCGAGTCATG  310
VRNA1-short_SNPs   GGTCGGCCTCATCATCTTCTCCACCAAGGGAAAGCTCTACGAGTTCTCCACCGAGTCATG  310

VRN-A1s            TGATATTTTGTTAGCTCCCAGTTACAAGTTAACTAATATATGGAGATCCTGGGCACGTAC  420
VRNA1-short_177in  TGATATTTTGTTAGCTCCCAGTTACAAGTTAACTAATATATGGAGATCCTGGGCACGTAC  370
VRNA1-short_SNPs   TGATATTTTGCTAGCTCCCAGTTACAAGTTAATTAATATATGGAGATCCTGGGCACGTAC  370

VRN-A1s            ATGTAAGCAGATCCTATCGACTTTCGTGGATCCGCTGATGGAATCACACCTCAGGATTTC  480
VRNA1-short_177in  ATGTAAGCAGATCCTATCGACTTTCGTGGATCCGCTGATGGAATCACACCTCAGGATTTC  430
VRNA1-short_SNPs   ATGTAAGCAGATCCTATCGACTTCCGTGGATCCGCTGATGGAATCGC-CCTCAGGATTTC  429

VRN-A1s            ATGGCATAGTCTTGATATGTCACGAACTGGTTGTTACTTTATTTATTTATTTTACACTGT  540
VRNA1-short_177in  ATGGCATAGTCTTGATATGTCACGAACTGGTTGTTACTTTATTTATTTATTTTACACTGT  490
VRNA1-short_SNPs   ATGGCATAGTCTTGATATGTCACGAATTAGTTGTTAGTTTATTTATTT-TTACATTGAGC  488

VRN-A1s            GTTCGCCCACCC------------------------------------------------  552
VRNA1-short_177in  GTTCGCCCACCCACAGGCCTGGCGCAGTGGTGAAGTCCTCCCCACTTGTGCCAAGAGGTC  550
VRNA1-short_SNPs   TTTGCCCATCCA------------------------------------------------  500

VRN-A1s            ------------------------------------------------------------  552
VRNA1-short_177in  CTGGGTTCGAACCAGCCTCTCTGCATTGCACTTTGCAGGGGTAAGACTAGGTTCCTATAA  610
VRNA1-short_SNPs   ------------------------------------------------------------  500

VRN-A1s            ------------------------------------------------------------  552
VRNA1-short_177in  TCCCTCCCCAGACCCCACCTTGTGTGGGAGCTTCTATGCACTGGGTCTGTCCTTTGTGTT  670
VRNA1-short_SNPs   ------------------------------------------------------------  500

VRN-A1s            ---------AGATGATAGAAATGTTATGTATTTAAAATATAATCTCACAGTCATTGTTGT  603
VRNA1-short_177in  CGCCCACCTAGATGATAGAAATGTTATGTATTTAAAATGTAATCTCACAGTCATTGTTGT  730
VRNA1-short_SNPs   ---------AGATGATAGAAATGTTATGTATTTAAAATGTAATCTCACAGTCATTGTTGT  551

VRN-A1s            TGGTATGGACCGTATGCCAAAAAGGGTTTTACCATGAAGTCTATTAATGAATAATTATTT  663
VRNA1-short_177in  TGGTATGGACCGTATGCCAAA---------------------------------------  790
VRNA1-short_SNPs   TGGTATGGACCGTATGCCAAA---------------------------------------  611

VRN-A1s            ATGTTAAAAAAAAAA                                               678
VRNA1-short_177in  ---------------                                               751
VRNA1-short_SNPs   ---------------                                               572
```

Figure S3: Alignment of *VRNA1*-short transcript variants (VRNA1-short_177in and VRNA1-short_SNPs) obtained by Sanger sequencing of cloned PCR products (VRNA1S_177inF/R) of cultivars Rescue and Pyrothrix 28 and published VRNA1-short sequence (VRN-A1s). SNPs are highlighted.

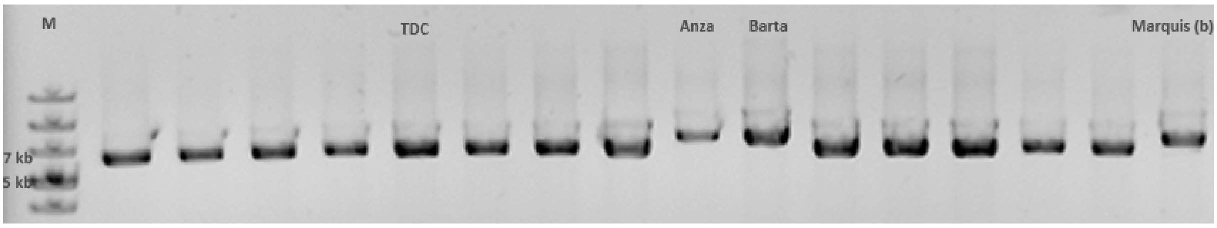

Figure S4: Agarose gel electrophoresis (0.8% agarose) of vrnB1_4F/R PCR products amplifying *VRN-B1* with and without 837 bp insertion. Anza, Barta and Marquis (01C0201025) = Marquis (b) – 6,8 kb, TDC – 6 kb. Lane M: GeneRuler 1 kb Plus DNA Ladder.

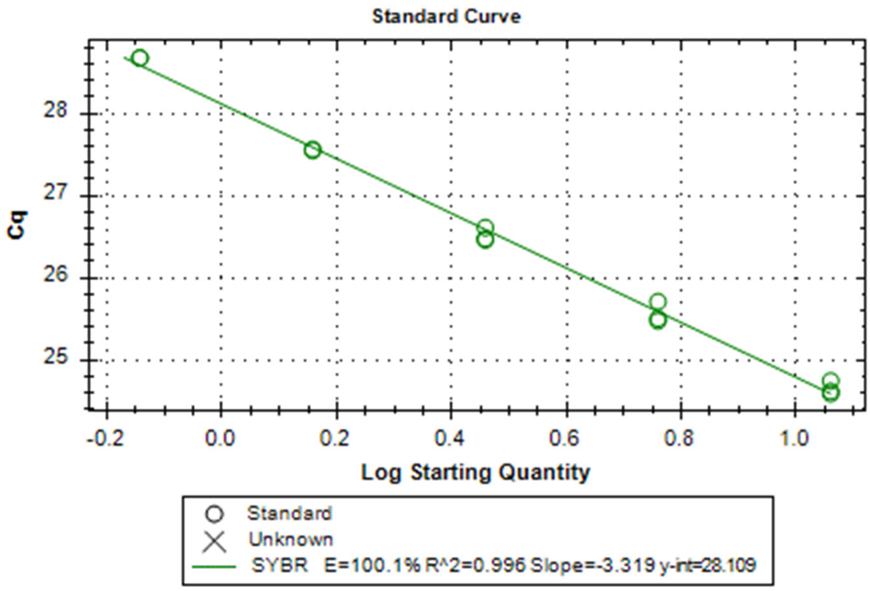

Figure S5: Standard curve of q.VRNB1 for 2-fold dilution series.

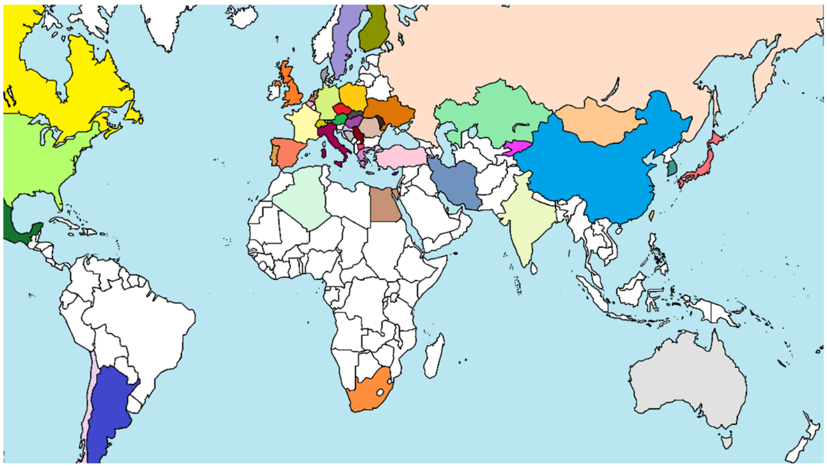

Figure S6: Geographical origin of 105 hexaploid wheat cultivars

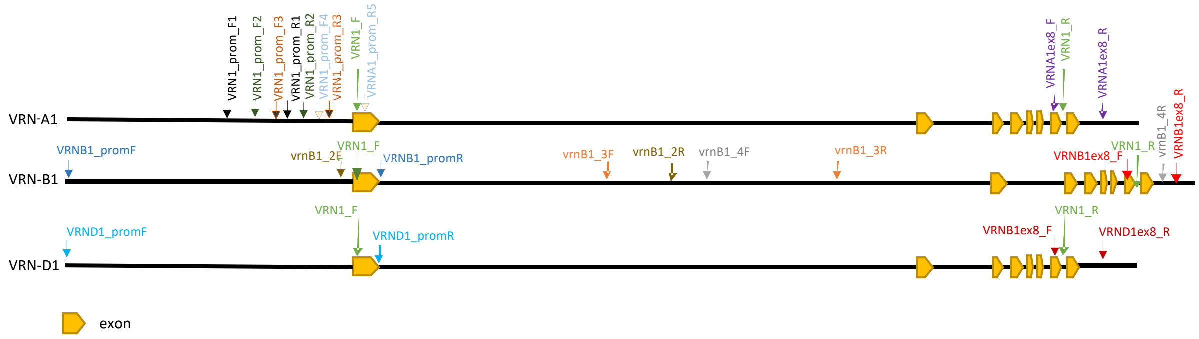

Figure S7: Overlapping amplicons used for *VRN-A1*, *VRN-B1* and *VRN-D1* sequencing.

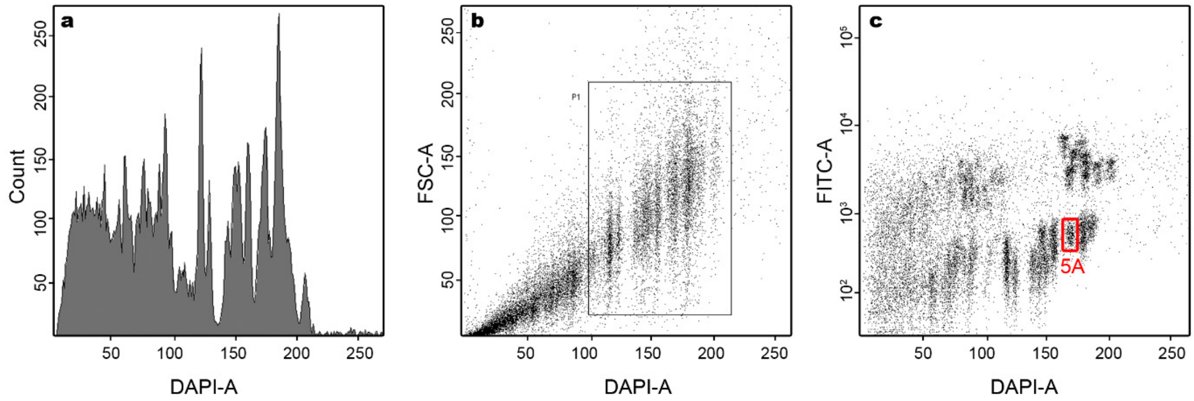

Figure S8: Flow sorting of chromosome 5A. (**a**) Monovariate flow karyotype of bread wheat. (**b**) Dot plot of relative DAPI fluorescence versus forward scatter intensity, P1 population delineates intact chromosomes and excludes chromatids, chromosome fragments and cell debris. (**c**) Bivariate scatter plot of relative DAPI fluorescence vs. relative Alexa488 fluorescence (in logarithmic scale) was used to discriminate individual chromosomes, sorting gate for chromosome 5A is highlighted in red.

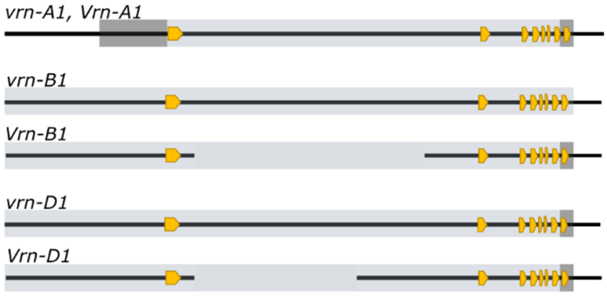

Figure S9 Schematic representation of recessive and dominant *VRN-1* alleles and their regions sequenced by different sequencing platforms. Polygons represent exons, dark grey rectangles represent regions sequenced by Sanger sequencing and light grey rectangles represent regions sequenced by Illumina iSeq. Oxford Nanopore sequencing was implemented for resolving the sequence of first *VRN-B1* intron in cultivars Anza, Barta and Marquis (01C0201025).
